# Autocrine VEGF signalling on M2 macrophages regulates PD‐L1 expression for immunomodulation of T cells

**DOI:** 10.1111/jcmm.14027

**Published:** 2018-11-20

**Authors:** Yin‐Siew Lai, Rika Wahyuningtyas, Shin‐Peir Aui, Ko‐Tung Chang

**Affiliations:** ^1^ Department of Biological Science and Technology National Pingtung University of Science and Technology Pingtung Taiwan; ^2^ Flow Cytometry Center Precision Instruments Center Office of Research and Development National Pingtung University of Science and Technology Pingtung Taiwan; ^3^ Departments of Fisheries and Marine Science University of Brawijaya Malang Indonesia

**Keywords:** autocrine, immunomodulation, macrophage, programmed death‐ligand 1, vascular endothelial growth factor

## Abstract

M2‐polarized macrophages, on one hand, can promote tumour vascularization by producing proangiogenic factors, such as vascular endothelial growth factor (VEGF). On the other hand, the expression of VEGF receptors (VEGFR) in this cell lineage was also reported. Although the function of VEGF/VEGFR axis plays a pivotal role in macrophages infiltration and angiogenesis, however, there is still lack of the direct evidence to show the role of VEGF as an autocrine operating in M2 macrophages, particularly for immunomodulation. In our study, we surprisingly discovered that M2 macrophages polarized by baicalin can simultaneously express VEGF and its receptors. Taking advantage of this unique culture system, we were able to investigate the biological activity of M2 macrophages in response to the autocrine VEGF milieu. Our results showed that the expression of programmed death‐ligand 1 (PD‐L1) on M2 macrophages was significantly up‐regulated in autocrine VEGF milieu. Through the blockade of autocrine VEGF signalling, PD‐L1 expression on M2 macrophages was dramatically down‐regulated. Furthermore, transplantation of PD‐L1^+^ M2 macrophage stimulated by autocrine VEGF into allogeneic mice significantly suppressed host CD4^+^/CD8^+^ T cells in the peripheral blood and increased CD4^+^ CD25^+^ regulatory T cells in the bone marrow. In conclusion, our findings provide a novel biological basis to support the current successful strategy using combined VEGF/PD‐1 signalling blockade in cancer therapy.

## 1 INTRODUCTION

Macrophages play a pivotal role in immune system and have functional plasticity depending on their phenotype of activation.^1^ Two types of macrophages, M1 and M2, were identified. M1 macrophages produce pro‐inflammatory cytokines such as tumour necrosis factor‐alpha (TNF‐α), interleukin‐12 (IL‐12), interleukin‐6 (IL‐6), and nitric oxide synthase (iNOS).^2^, ^3^ In contrast, the “alternatively activated” anti‐inflammatory M2 macrophages,^3^ can be induced by TH2 factors such as interleukin‐4 (IL‐4) and interleukin‐13 (IL‐13) to secrete interleukin‐10 (IL‐10) and transforming growth factor‐beta (TGF‐β) for reducing the inflammatory response and stimulating tissue repair in the late inflammatory response.^4^ In addition, M2 macrophages have high phagocytosis capacity, producing extracellular matrix components, angiogenic and chemotactic factors.^5^ Meanwhile, M2 phenotypes were subdivided into M2a, M2b, M2c according to similarities and differences among activation by IL‐4 (M2a), by immune complex plus toll‐like receptor (TLR) ligands (M2b), and by IL‐10 and glucocorticoids (M2c).^6^ M2d macrophages are the other subtype of M2 macrophages induced by costimulation with TLR and adenosine A2A receptor agonists.^7^ Besides, M2d macrophages treated with the combination of lipopolysaccharide (LPS) and 5′‐*N*‐ethylcarboxamidoadenosine expressed high levels of vascular endothelial growth factor (VEGF), IL‐10, and iNOS, low levels of TNF‐α and IL‐12, and mildly elevated levels of arginase‐1.^8^ Previous study investigated that additional VEGF‐induced angiopoietin‐2 (ANGPT2) can up‐regulate programmed death‐ligand 1 (PD‐L1) expression on M2‐polarized macrophages derived from human peripheral blood mononuclear cells (PBMC).^9^ Another study showed that a fraction of monocytes/macrophages in the peri‐tumoural stroma, while expressing surface PD‐L1 molecules, effectively suppressed tumour‐specific T cell immunity and contributed to the tumours progression in vivo; the effect could also be reversed by blocking PD‐L1 on those monocytes.^10^ To the best of the current findings, whether VEGF not only acts as a paracrine, but also an autocrine for M2 macrophage to regulate PD‐L1 expression and T cell immunomodulation remains unknown.

Recently, the large‐scale randomized clinical trials demonstrated excitingly that anti‐VEGF enhanced the efficacy of PD‐L1 blockade in cancer therapy. For instance, simultaneous blockade of programmed cell death protein 1 (PD‐1) and VEGF receptor 2 (VEGFR2) dramatically decreases the tumour size and tumour neovascularization in vivo.^11^ PD‐1 blockade combined with ANGPT2 and VEGF‐A blockade improves the anti‐tumoural activity by decreasing the mean tumour volume and weight.^12^ Furthermore, in the experimental mice, the combination of anti‐angiogenic/anti–PD‐L1 therapy impaired tumour regrowth and resulted in a low tumour burden.^13^ Although a possible affiliation of VEGF and PD‐L1 was implied by its synergistic effect in tumour progression, the biological interpretations were restricted, mainly addressing on the independent role of VEGF and PD‐L1 in angiogenesis and T cell suppression, respectively.

In our present study, we surprisingly found that baicalin, one of flavone glucoside extracted from the roots of *Scutellaria baicalensis,* not only can polarize macrophages toward VEGF‐secreting M2d macrophages, but also promote their expression of VEGF receptor simultaneously. We therefore were capable of creating an isolated milieu to investigate the biological activity of M2 macrophages in response to autocrine VEGF, especially for immunomodulation.

## 2 MATERIALS AND METHODS

### 2.1 Cell lines

RAW 264.7 cells (murine macrophage cell line) were purchased from American Type Culture Collection (ATCC, Manassas, VA, USA). Cells were cultured in 90% Dulbecco's Modified Eagle's Medium (Corning, Manassas, VA, USA) with 10% Foetal Bovine Serum (Hyclone, Logan, UT, USA) and grown under standard cell culture conditions in 5% CO_2_ at 37°C to reach confluence of 50%‐60% before subjecting to any further experiment. Medium was refreshed every 24 hours.

### 2.2 Macrophage polarization

5 × 10^5^ cells/mL of RAW 264.7 cells were seeded in cultures overnight before treatment. Cells were incubated with LPS (1 μg/mL) (Sigma, St. Louis, MO, USA) for 2 hours, IL‐4 (10 ng/mL) (CELL Guidance System, St. Louis, MO, USA) for 24 hours and baicalin (50 μmol/L) (Tokyo Chemical Industry, Tokyo, Japan) for 24, 48, and 72 hours. Cells were analysed by microscopy for morphological studies on 24, 48, and 72 hours.

### 2.3 Mice

Six‐ to 10‐week‐old adult female C57BL/6J mice were purchase from National Laboratory Animal Center (Taipei, Taiwan) and housed in a clean conventional animal facility at 22°C with 12‐h light/dark cycle. Sterilized food and water were freely accessible in their cage. The protocol was approved by the Institutional Animal Care and Use Committee of College (IACUC) of Veterinary Medicine at National Pingtung University of Science and Technology.

### 2.4 Monocyte‐to‐macrophage differentiation and polarization

Bone marrow mononuclear (BMMNC) cells were collected by flushing the femurs and tibias from mice with PBS (J.T Baker, Phillipsburg, NJ, USA) in pH 7.4, containing 0.5% bovine serum albumin (BSA) (Sigma). The mononuclear cells were obtained after Ficoll‐paque™ PLUS (GE Healthcare, Uppsala, Sweden) density gradient centrifugation. Briefly, M1 was induced by incubating isolated mononuclear cells with M‐CSF (50 ng/mL; PEPROTECH, Rocky Hill, NJ, USA) for 7 days in RPMI 1640 (Corning, Manassas, VA, USA) supplemented with 10% foetal calf serum (PAA, Pasching, Austria), followed by LPS (1 μg/mL) treatment for 2 hours. M2 was induced by incubating mononuclear cells with 50 ng/mL M‐CSF for 7 days, followed by polarization with 10 ng/mL IL‐4 (CELL Guidance System, St. Louis, MO, USA) or 50 μmol/L baicalin (Tokyo Chemical Industry) for 24 hours. The results from BMMNC are only presented in Data S2 and S3.

### 2.5 Gene expression

Total cellular RNA was isolated by lysing the cells (1 × 10^6^) in 1 mL of Tripure Isolation Reagent (Roche Life Science, Mannheim, Germany). RNA was treated with chloroform, centrifuged (12 000 *g*, 15 minutes, 4°C), and finally precipitated with ethanol. The RNA was resuspended in RNase‐free water and the RNA concentration was determined by light absorbance at 260 nm (MaestroNano, Maestrogen, Taiwan). Total RNA (1 μg) was used in the reverse transcription (RT) reaction with 0.5 μg of oligo dT_15_ (Promega, Madison, WI, USA), 0.5 mmol/L of each of the four deoxynucleotide triphosphates, 25 mmol/L MgCl_2_, and 1 μL of GoScript™ Reverse Transcriptase (Promega) according to the manufacturer's instructions. The real time PCR was performed using KAPA SYBR^®^ FAST qPCR Master Mix (2X) Kit (KAPA Biosystem, Wilmington, DE, USA) according to the manufacturer's protocol. The primer sequences are listed in Table 1. Ensured all reaction components properly thawed and mixed.

**Table 1:** Primer sequence

Primer	Sequencing	References
*TNF‐*α	Forward: 5′‐TTGACCTCAGCGCTGAGTTG‐3′	36
	Reverse: 5′‐CCTGTAGCCCACGTCGTAGC‐3′	
*IL‐6*	Forward: 5′‐GAGGATACCACTCCCAACAGACC‐3′	37
	Reverse: 5′‐AAGTGCATCATCGTTGTTCATACA‐3′	
*IRF5*	Forward: 5′‐GTTGCCTTTGACGGACCTA‐3′	38
	Reverse: 5′‐GGCCCACTCCAGAACACCT‐3′	
*Arginine 1*	Forward: 5′‐GACAGCAGAGGAGGTGAAGAGT‐3′	39
	Reverse: 5′‐GGTAGTCAGTAACTGGCTTATG‐3′	
*IL‐10*	Forward: 5′‐TTTGAATTCCCTGGGTGAGAA‐3′	40
	Reverse: 5′‐CTCCACTGCCTTGCTCTTATTTTC‐3′	
*IRF4*	Forward: 5′‐CTCTTCAAGGCTTGGGCATT‐3′	41
	Reverse: 5′‐TGCTGCTTTTTTGGCTCCCT‐3′	
*VEGF‐A*	Forward: 5′‐TGCAGATTATGCGGATCAAACC‐3′	42
	Reverse: 5′‐TGCATTCACATTTGTTGTGCTGTAG‐3′	
*VEGF‐B*	Forward: 5′‐TCTCGCCATCTTTTATCTCCCAG‐3′	43
	Reverse: 5′‐CAGAACCCAAATCCCGTTATTG‐3′	
*VEGF‐D*	Forward: 5′‐ATGGCGGCTAGGTGATTCC‐3′	44
	Reverse: 5′‐CCCTTCCTTTCTGAGTGCTG‐3′	
*VEGFR‐1*	Forward: 5′‐CCTCACTGCCACTCTCATTGTA‐3′	29
	Reverse: 5′‐ACAGTTTCAGGTCCTCTCCTT‐3′	
*VEGFR‐2*	Forward: 5′‐GGAAGCTCCTGAAGATCTGT‐3′	45
	Reverse: 5′‐GAGGATATTTCGTGCCGC‐3′	
*VEGFR‐3*	Forward: 5′‐GTCCCTCTACTTCCAACTGC‐3′	32
	Reverse: 5′‐CACTCCTCCTCTGTGACTTTGAG‐3′	
*PD‐L1*	Forward: 5′‐TGCTGCATAATCAGCTACGG‐3′	46
	Reverse: 5′‐GCTGGTCACATTGAGAAGCA‐3′	
β*‐actin*	Forward: 5′‐AGACTTCGAGCAGGAGAT‐3′	47
	Reverse: 5′‐ATGCCACAGGATTCCATAC‐3′	

### 2.6 Flow cytometry

Flow cytometry (BD Biocsiences, San Jose, CA, USA) was used to measure the expression of surface protein on cells and the analysis was performed using BD FACSDiva Software (BD Biocsiences) and FlowJo Software (Tree Star, Inc., Ashland, OR, USA). Briefly, harvest, wash and adjust the cells in the suspension to a concentration of 1 × 10^6^ cells/mL on ice. Cells were centrifuged sufficiently; discarded supernatant and stained in 12 × 75 mm^2^ polystyrene round bottom tubes with fluorescence‐conjugated antibody (FITC‐CD11b [0.25 μg], FITC‐CD86 [1 μg], APC‐CD206 [0.5 μg], PE‐VEGFR2 [CD309, 1 μg], APC‐PD‐L1 [CD274, 0.25 μg], APC/Cy7‐PD‐1 [CD279, 0.1 μg], PerCP‐CD3 [0.25 μg], APC‐CD4 [0.25 μg], PE‐CD8 [0.25 μg], and FITC‐CD25 [0.1 μg]) (Biolegend, San Diego, CA, USA). Incubate for at least 30 minutes in dark at room temperature. Wash the cells 3× by centrifugation at 400 *g* for 5 minutes and resuspend the cells in 500 μL to 1 mL of cold PBS. Keep the cells in the dark on ice or at 4°C in a fridge until the scheduled time for analysis.

### 2.7 Enzyme‐linked immunosorbent assay (ELISA)

Cell‐free supernatants were collected and stored at −20°C until assayed for cytokine levels. The amount of VEGF proteins in the supernatants was determined using mouse VEGF ELISA Kit (Boster Biological Technology Co Ltd, Pleasanton, CA, USA), according to the manufacturer's instructions. Read the absorbance of each well at 450 nm in the microplate by EZ Read 400 Microplate Reader (Biochrom, Cambridge, UK).

### 2.8 Evaluation of allostimulatory activity of PD‐L1^+^ M2 macrophages in vivo

Nine to 12 weeks old female C57BL/6 mice were divided into two groups. For PD‐L1^hi^ group, mice were injected through retro‐orbital plexus with 1 × 10^6^/100 μL RAW 264.7‐derived M2 macrophages polarized by 50 μmol/L baicalin (48 hours) on day 0, day 7, and day 14, respectively. In PD‐L1^lo/−^ group, RAW cells without stimulation by baicalin were injected via the same way. The mice were killed on day 19 after transplantation and the mononuclear cells from spleen, peripheral blood, and bone marrow were collected for flow cytometry analysis of T cells composition.

### 2.9 Collection of mononuclear cells from spleen, peripheral blood, and bone marrow in mice

The whole spleen was squeezed by glass grinder and the dissociated cells were filtered through a 40 μm cell strainer (BD Bioscience). 50 μL heparinized blood was lysed with 3 mL RBC lysis buffer, shaking at 37°C, 250 rpm for 15 minutes to remove red blood cells. Total bone marrow cells were collected from femur and tibia of mice by flushing with 1 mL PBS twice through 25G syringe.

### 2.10 Statistical analysis

All results were collected from at least three independent experiments and data were presented as mean ± SEM. Statistical significance of pairwise differences among three or more groups were determined using one‐way analysis of variance (ANOVA) followed by LSD test. Statistical analysis was performed with SPSS for Windows (Version 20.0; SPSS Inc., Chicago, IL, USA) and Graphpad Prism 7 (GraphPad Software, Inc, La Jolla, CA, USA). *P*‐value less than 0.05 were considered as statistically significant.

## 3 RESULTS

### 3.1 M2 macrophages polarization by baicalin

**Figure 1:**
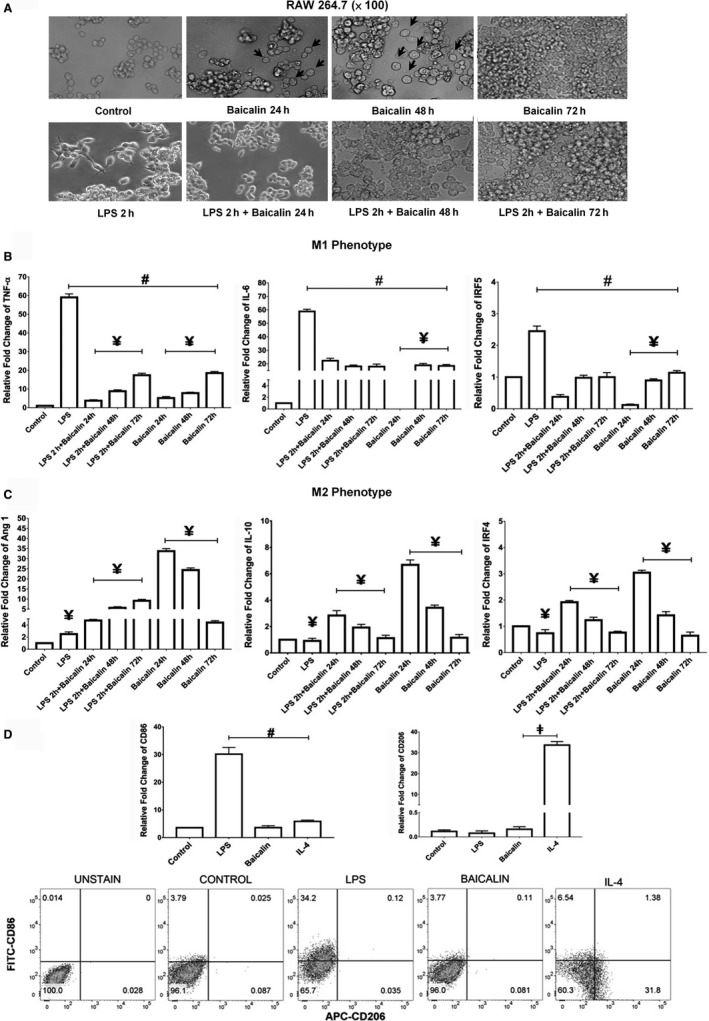
Baicalin promoted M2 macrophage polarization. (A) Morphological characteristics of macrophages observed by microscopy (magnification ×100), (B, C) gene expression analysis of M1 and M2 macrophages by real time PCR, (D) the expressions of surface protein CD86 and CD206 in macrophages by flow cytometry. Data were determined by mean ± SEM, n = 6, one of six representative experiments is shown. ^#^ *P* < 0.05 compared with LPS; ^¥^ *P* < 0.05 compared with 24 h treatment of baicalin; ^‡^ *P* < 0.05 compared with IL‐4. LPS, lipopolysaccharides

To access the polarization of M2 macrophages by baicalin, we used RAW 264.7 macrophage cell line. We further characterized the phenotype of macrophages induced by baicalin vs LPS. The results showed the significant differences in morphology between M1 and M2 macrophages polarized by LPS and baicalin, respectively, after 24 hours (Figure 1A). Macrophages polarized by baicalin showed atypical round shape and larger size than that induced by LPS. Gene expression profiling showed that M1 macrophage phenotypes were significantly down‐regulated (Figure 1B), and M2 macrophage phenotypes were correspondingly up‐regulated after the treatment of baicalin (Figure 1C). Interestingly, M1 macrophages polarized by LPS can easily be switched to M2 macrophages following baicalin induction (Figure 1C). Our results were in parallel to the previous study.^14^ Meanwhile, FACS analysis of CD206, a typical M2 macrophages marker, was not expressed dominantly on M2 macrophages polarized by baicalin compared with that polarized by IL‐4 (Figure 1D). Thus, according to CD206 and CD86 expression, it was so firmly to explain that M2 macrophages polarized by baicalin were not belonging to M2b character.^7^, ^8^

### 3.2 M2 macrophages polarized by baicalin expressed VEGF‐A and its receptors

**Figure 2:**
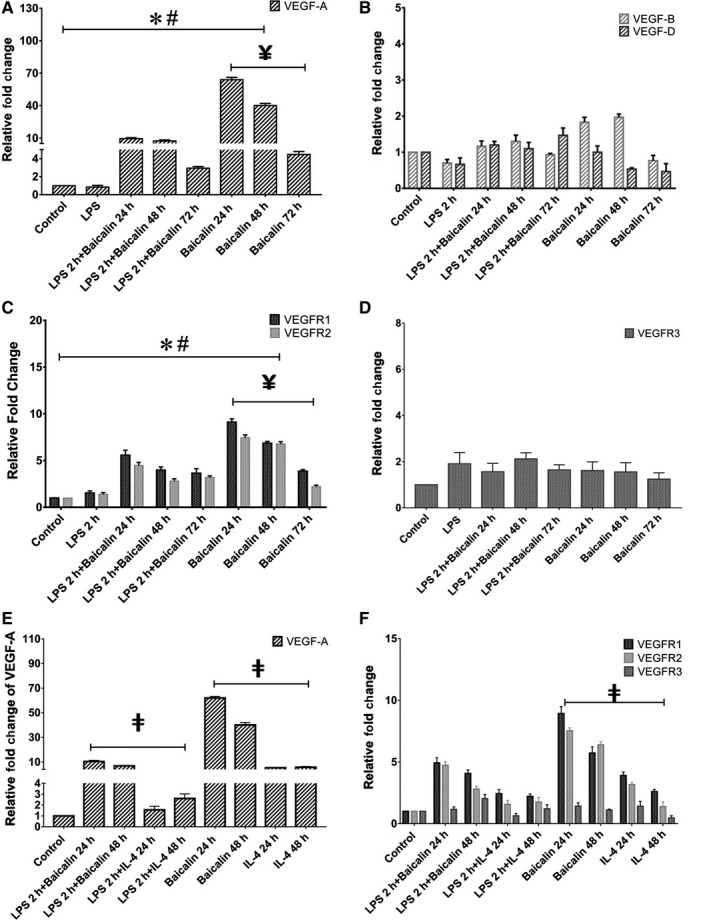
M2 macrophages polarized by baicalin expressed *VEGF‐A* and its receptors. A, B, The expression of *VEGF* family. C, D, The expression of *VEGFR* family. E, F, The comparison of the mRNA expression of *VEGF‐A* and *VEGFR* in M2 macrophages polarized by baicalin and IL‐4. Data were determined by mean ± SEM, n = 4. *P < 0.05 compared with control group; ^#^ *P* < 0.05 compared with LPS; ^¥^ *P* < 0.05 compared with 24 h treatment of baicalin; ^‡^ *P* < 0.05 compared with IL‐4. VEGF, vascular endothelial growth factor; LPS, lipopolysaccharide; IL‐4, interleukin‐4

We further evaluated the gene transcriptional expression of VEGF/VEGFR family on M2 macrophages polarized by baicalin. Our results showed that the highest expression of *VEGF‐A* appeared after 24 hours of the treatment (Figure 2A). In contrast, the expression of *VEGF‐B* and *VEGF‐D* were not changed (Figure 2B). Unexpectedly, the expression of VEGF receptors, *VEGFR1* and *VEGFR2* but not *VEGFR3,* were also significantly up‐regulated after 24 hours of the treatment (Figure 2C,D). Once again, the gene transcripts indicated above could easily be up‐regulated in LPS‐polarized M1 macrophages followed by baicalin induction. In comparison with M2 macrophages polarized by IL‐4, the significantly lower expression of *VEGF‐A* and its receptors *VEGFR1* and *VEGFR2* were found (Figure 2E,F). We also confirmed the similar results in bone marrow derived M2 macrophages polarized by baicalin (Figure S2).

### 3.3 Autocrine VEGF milieu of M2 macrophages

**Figure 3:**
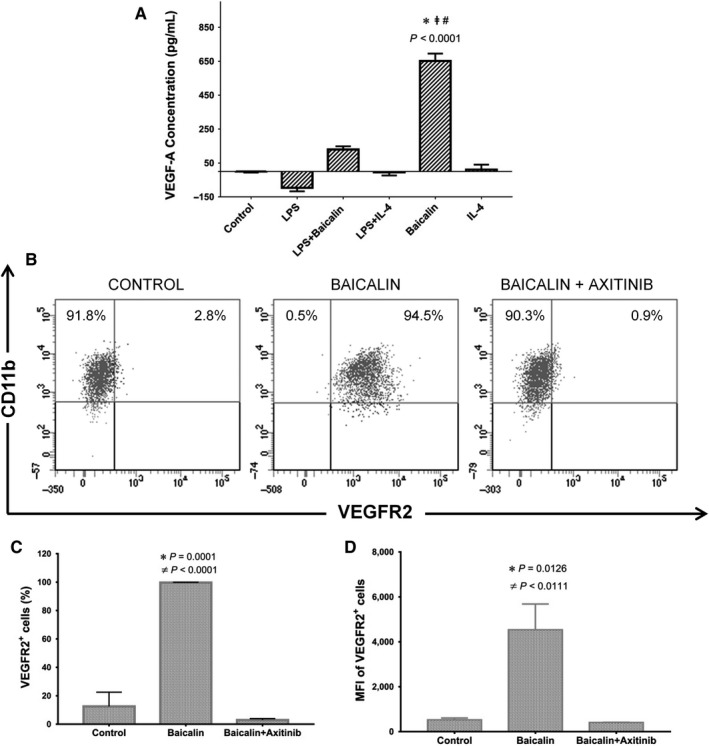
Autocrine VEGF milieu of M2 macrophage interfered by VEGF receptor inhibitor. A, The secreted VEGF‐A protein in conditioned media were measured. B, C, The percentage of M2 macrophages expressing VEGF‐R2. D, The mean fluorescence intensity (MFI) of VEGF‐R2 on M2 macrophages. The Data were determined by mean ± SEM, n = 3. *P < 0.05 compared with control group; ^#^ *P* < 0.05 compared with LPS; ^‡^ *P* < 0.05 compared with IL‐4; ≠*P* < 0.05 compared with axitinib. VEGF, vascular endothelial growth factor; LPS, lipopolysaccharide; IL‐4, interleukin‐4

By the simultaneous expression of *VEGF‐A* and its receptors, *VEGFR1* and *VEGFR2*, in M2 macrophages polarized by baicalin, we hypothesized that autocrine VEGF milieu was established in this culture system. To test this hypothesis, we examined the secreted form of VEGF‐A in conditioned media as well as the surface protein of VEGFR2 on CD11b^+^ M2 macrophages (Figure 3A,B). Expectedly, the protein expressions of VEGF‐A and VEGFR2 were significantly increased comparing to the control group (Figure 3A,C). Moreover, by using axitinib as the blockade of VEGF receptor dramatically decreased the expression of VEGFR2 both in the whole population of M2 macrophages and in a single cell (Figure 3B,D). The effect of axitinib was confirmed from the prior study using retinal pigment epithelial cells and human umbilical vein endothelial cells.^15^ It is worth noting that the untreated cells in control group did not express VEGF‐A (Figure 3A) and only 2.8% of those cells expressed VEGFR2 (Figure 3B). Therefore, the effect of axitinib on untreated control cells seems not be curious to be explored.

### 3.4 Autocrine VEGF signalling up‐regulated PD‐L1 expression in M2 macrophages

**Figure 4:**
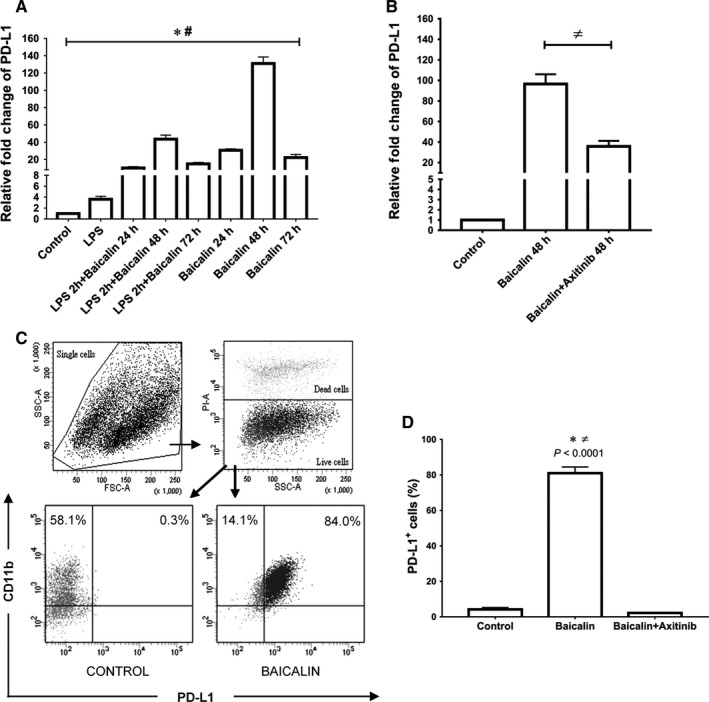
Autocrine VEGF signalling up‐regulated PD‐L1 expression on M2 macrophages. A, The expression of *PD‐L1* in M2 macrophages was significantly up‐regulated after 48 h treatment of baicalin. B, The expression of *PD‐L1* in M2 macrophages was significantly suppressed by the blockade of autocrine VEGF signalling. C, Flow cytometry analysis of PD‐L1 expression. D, The percentage of PD‐L1^+^ M2 macrophage was increased upon autocrine VEGF signalling. Data were determined by mean ± SEM, n = 4, *P < 0.05 compared with control group; ^#^ *P* < 0.05 compared with LPS; ≠*P* < 0.05 compared with axitinib. VEGF, vascular endothelial growth factor; PD‐L1, programmed death‐ligand 1; LPS, lipopolysaccharide

Owing to the prior study that additional VEGF‐induced angiopoietin‐2 up‐regulated PD‐L1 expression on PBMC‐derived M2 macrophages, we therefore assessed both the transcriptional and translational expression of PD‐L1 in M2 macrophages grown upon our established autocrine VEGF milieu. Interestingly, the expression of *PD‐L1* was significantly increased over 100‐fold changes compared with the control group (Figure 4A). Moreover, the blockade of VEGF receptor signalling significantly down‐regulated the expression of *PD‐L1* in M2 macrophages (Figure 4B). The results showed that PD‐L1 surface protein expression on CD11b^+^ M2 macrophages was in parallel with the gene transcriptional outcome described above (Figure 4C,D). Furthermore, PD‐L1 surface protein expression was also up‐regulated on bone marrow derived CD11b^+^ M2 macrophages in baicalin‐induced VEGF culture milieu (Figure S2).

### 3.5 Allostimulatory activity of PD‐L1^+^ M2 macrophages in vivo

**Figure 5:**
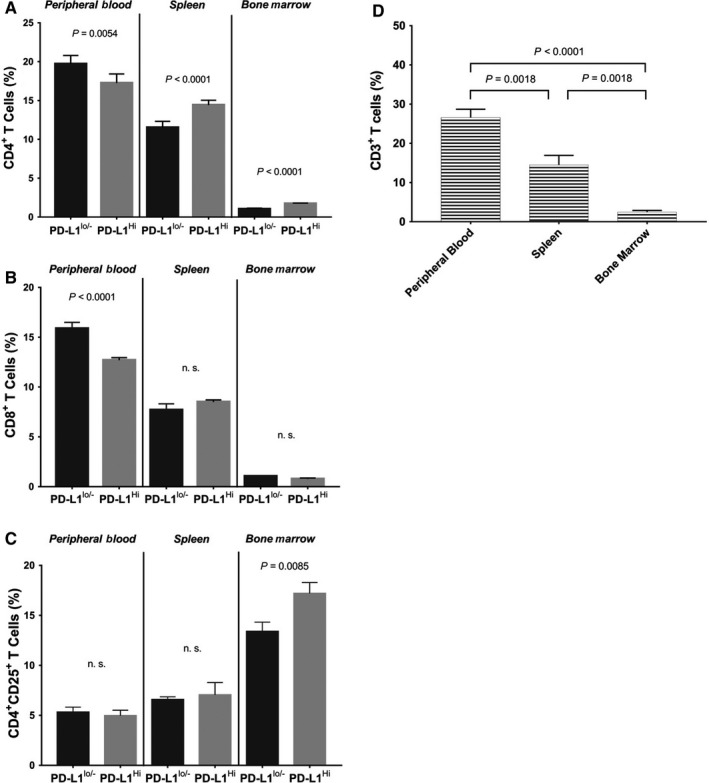
Allostimulatory activity of PD‐L1^+^ M2 macrophages in vivo. The allostimulatory activity of PD‐L1^+^ M2 macrophages was determined by measuring the cell frequency of host T cell after transplantation. A, The percentage of CD4^+^ T cells. B, The percentage of CD8^+^ T cells. C, The percentage of CD4^+^ CD25^+^ Tregs cells, in peripheral blood, spleen, bone marrow, respectively. D, The percentage of peripheral blood CD3^+^ total T cells in mice before transplantation. Data were determined by mean ± SEM, n = 5. *P* value was calculated by one‐way ANOVA with Tukey test. PD‐L1, programmed death‐ligand 1

According to Sakhno et al's study, B7‐H1^+^ (PD‐L1)‐ M2 macrophages prohibited allogeneic T cell proliferating activity in mix lymphocyte culture that was associated with the higher numbers of apoptotic T cells through PD‐1/PD‐L1 pathway.^16^ To verify in vivo functionality of PD‐L1^+^ M2 macrophages stimulated by autocrine VEGF, we focused on their immunomodulatory capacity in allogeneic mice model. Flow cytometry analysis firmly showed that by transplantation of ~84% vs ~4% PD‐L1^+^ M2 macrophages, the cell frequency of host CD8^+^ and CD4^+^ T cells in peripheral blood were significantly reduced 19 days post‐transplantation (Figure 5A,B). Interestingly, a group of CD4^+^CD25^+^ regulatory T cells was significantly increased in bone marrow after transplantation (Figure 5C). According to previous literature,^17^ it is worth noting that cell frequency of CD8^+^ and CD4^+^ cells in the peripheral blood of normal mice were 7%‐10% and 8%‐12%, respectively. Our data in parallel showed that CD3^+^ total T cell was less than 26.6% in the peripheral blood of mice before transplantation (Figure 5D). However, the cell frequency of CD8^+^ and CD4^+^ T cells in the peripheral blood of host mice in PD‐L1^lo/−^ group were slightly higher (~16% and 19.7%, respectively) than normal. This result indicated that a gentle graft vs host (GVH) immune‐response by transplantation of total 1 × 10^6^ RAW 264.7 cells (Balb/c) into C57BL/6J mice was possibly provoked. In contrast with PD‐L1^lo/−^ group, the reactivity of GVH was significantly alleviated in PD‐L1^hi^ group (CD8, 12%; CD4, 17%). Therefore, the regulation of T cells functions upon the interaction with PD‐L1^+^ M2 macrophages stimulated by autocrine VEGF was truly confirmed.

## 4 DISCUSSIONS

**Figure 6:**
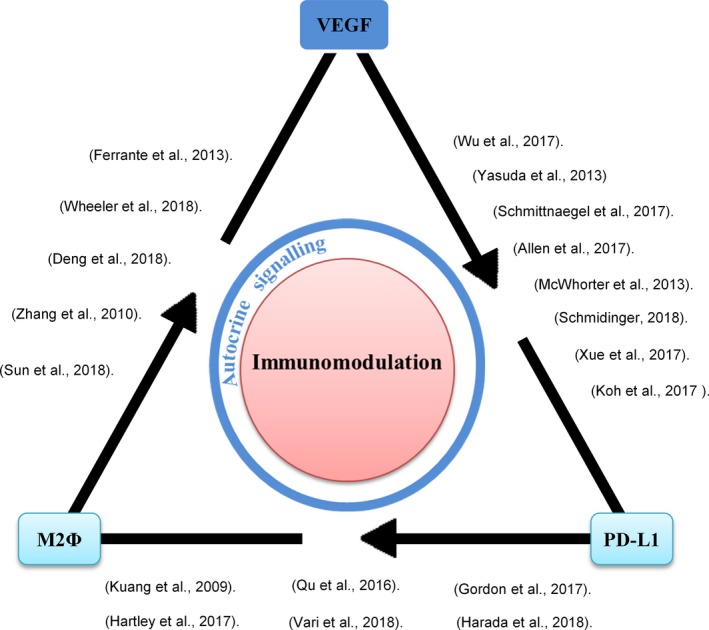
Trilateral relationship among M2 macrophages, autocrine VEGF and PD‐L1 expression for their role of immunomodulation. The bilateral co‐relations were indicated by the previous studies. Autocrine VEGF that regulates the expression of PD‐L1 on M2 macrophages was proved in our study. PD‐L1^+^ M2 macrophages participate in T cell immunomodulation. VEGF, vascular endothelial growth factor; PD‐L1, programmed death‐ligand 1

According to our results, we attempt to orchestrate a triangle relationship among M2 macrophages, autocrine VEGF/VEGFR and PD‐L1 expression for their role in immunomodulation (Figure 6). At present, the evidence were merely restricted by their bilateral relationship, respectively; and in most of the circumstances, VEGF was identified to play a role as a paracrine factor or stimulus for M2 macrophages’ development. In order to strengthen our finding that M2 macrophages can autonomously regulate PD‐L1 expression by operating autocrine VEGF stimulation or loop, we therefore highlight those important evidence so far about their bilateral corelations, including.

### 4.1 Corelation of VEGF and PD‐L1

There are few but important direct evidence to support the co‐relation between VEGF and PD‐L1. The recent study on clear cell renal cell carcinoma showing PD‐L1 expression by immunohistochemistry staining was associated with VEGF expression that makes adverse pathological features in patients.^18^ Another study emphasized that added ANGPT2 promoted PD‐L1 expression on CSF1, IL‐10, and IL‐4 activated M2 macrophages.^9^ Although these findings strongly suggested that tumour cells or M2 macrophages were capable of receiving VEGF signalling for PD‐L1 up‐regulation. However, the results were unable to elucidate whether M2 macrophages can deliver VEGF autonomously for their regulation of PD‐L1 expression. In contrast, our study successfully indicated that M2 macrophages were able to signal autocrine VEGF for PD‐L1 regulation. On the other hand, the indirect evidence were supported by many of the clinical reports. For example, Xue et al proved that positive VEGF expressions around the vessels were more significant frequently observed in the PD‐L1 positive group of high‐grade glioma and Hodgkin lymphoma.^19^, ^20^ In addition, the indirect evidence can be seen in experimental animals that PD‐1 blockade improved the anti‐tumoural activity of combined ANGPT2 and VEGFA blockade, which decreased the mean volume and weight of tumour and increased proportions of IFNɤ^+^ T and NK cells.^12^ This was also in parallel with another study showing that blockade of PD‐1 and VEGFR2 decreased the tumour size and tumour neovascularization in rodent.^11^ Furthermore, PD‐L1 was up‐regulated in mouse tumours relapsing from antiangiogenic therapy.^13^

### 4.2 Corelation of M2 macrophages and PD‐L1

Tumour‐associated macrophages (TAM) of the M2 phenotype expressing PD‐L1 are also one of the most inspiring topics that has been discussing among researchers. A previous study showed that PD‐L1^+^ monocytes were accumulated in the peritumoural stroma area of cancers and increased with tumour progression. These activated PD‐L1^+^ monocytes suppressed tumour‐specific T cell proliferation, cytokine production, and cytotoxic potential in vitro and also fostered tumour growth in NOD/SCID mice bearing human tumours.^10^ Corresponding to TAM study showed that the tumour‐conditioned media strongly induced PD‐L1 expression on bone marrow‐derived monocytes mediated by TNF‐α.^21^ Besides that, a recent study showed that CD14^+^CD68^hi^CD163^hi^ intratumoural monocyte/M2 macrophages had pronounced dual protein expression of PD‐L1/PD‐L2 in both classical Hodgkin lymphoma (cHL) and diffuses large B‐cell lymphoma (DLBCL).^22^ Furthermore, other investigators have reported the increase in PD‐L1‐expressing CD68^+^ macrophage in circulating blood of ovarian cancer patients, and the expression of PD‐L1 on TAM promoted apoptosis of T cells via interaction with PD‐1 on CD8^+^ T cells.^23^

For cancer cells that express PD‐L1 to affect macrophage activity, Gordon and the team found that PD‐1 expression on TAMs correlates with phagocytosis inhibition and total in vivo phagocytosis levels.^24^ The other study has demonstrated that CD163^+^ M2‐like macrophage infiltration is highly associated with PD‐L1 expression in gastric adenocarcinomal cells.^25^

### 4.3 Corelation of M2 macrophages and VEGF

The previous study proved that VEGF can polarize THP‐1‐derived macrophages toward the M2 phenotype and enhanced macrophage migration.^26^ The similar result highlighting a novel function of both recombinant VEGF‐C protein and tumoural VEGF‐C could efficiently enhance migration of murine macrophages RAW 264.7 cell. Tumoural VEGF‐C also acted paracrinely to induce macrophage recruitment, and resultantly promoted clinical nonsmall cell lung cancer cell metastasis.^27^ Moreover, a decrease in TAM (CD45^+^, CD11b^+^, F4/80^+^) was observed upon axitinib treatment in both subcutaneous MC38 and LLC1 tumour cells.^28^

On the other side, a study identified that baicalin can increase VEGF expression through the activation of the ERRα pathway in U251 human glioma cells and implicated the participation of macrophages in angiogenesis.^29^ Again, TGF‐β1 promotes VEGF secretion in bone marrow derived macrophages and in oral squamous cell carcinoma TAM was reported.^30^

We address the function of baicalin for initiating M2 macrophages polarization and autocrine VEGF operation. The previous study showing that human PBMC treated with 1 mM baicalin can significantly enhance the IFN‐γ secretion in the cell culture supernatants.^31^ Additionally, PBMC from ulcerative colitis patients intervened by baicalin were obviously elevated the levels of IL‐10.^32^ In our study, we also found an increased level of *IL‐10* expression in RAW 264.7 and in bone marrow derived M2 macrophages (data not shown). Besides, the previous study suggested that increased levels of TNF‐α, IL‐1β, and IL‐6 was reversed by baicalin treatment in damaged colon tissues.^33^ Upon on those findings, we hypothesize that autocrine VEGF operation by transient activation of monocytes/macrophages highly depends on the soluble factors, including IFN‐γ, IL‐10 and the proportion of proinflammatory cytokines in the milieu. In combination with our finding and the evidence demonstrated that elevated PD‐L1 expression on TAM can be inhibited by anti‐IL‐10 antibody,^34^ therefore, the PD‐L1 expression on TAM regulated by autocrine VEGF potentially existed.

Hereby, our findings enhance overall understanding of autocrine VEGF that participates in the regulation of PD‐L1 expression on M2 macrophages for immunomodulation. We not only construct the actual trilateral relations among M2 macrophages, autocrine VEGF and PD‐L1 expression for their role of immunomodulation, but also support a theory in biological aspect to explain how anti‐VEGF signalling turns to be a main character to enhance the effect of PD‐1 blockade in cancer therapy. Additionally, it highlights the value for the development of inhibitors targeting hypoxia‐inducible factor 1 and 2 in TAMs.^35^ Further investigation is needed to examine whether PD‐L1^+^ M2 macrophages stimulated by autocrine VEGF can be potent for immunotherapy of autoimmune diseases.

## CONFLICT OF INTEREST

The authors have no conflicts of interests to declare.

## AUTHOR CONTRIBUTIONS

K.T.C. conceived and designed the experiments. K.T.C. wrote the manuscript. R.W. performed q‐PCR and ELISA. Y.S.L. performed flow cytometry. Y.S.L and S.P.A. performed the animal experiments. Y.S.L., R.W., and K.T.C. analysed the data. K.T.C. contributed reagents/materials/analysis tools. Y.S.L., S.P.A. proofread the manuscript. K.T.C. approved the final version.
