# Supplementary figures and images for: Autocrine VEGF signalling on M2 macrophages regulates PD‐L1 expression for immunomodulation of T cells
Source: J Cell Mol Med. 2018 Nov 20;23(2):1257–67. doi: 10.1111/jcmm.14027 (PMC6349155; doi:10.1111/jcmm.14027)

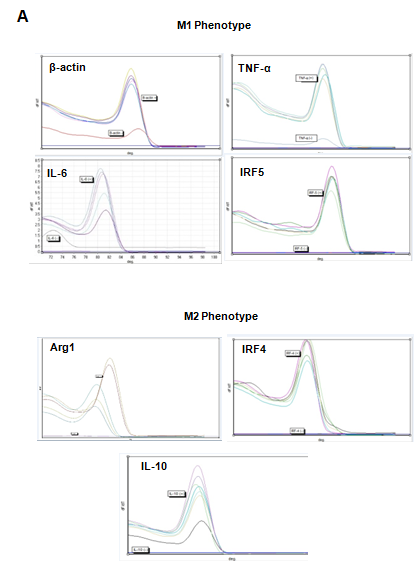

Supplement: Supplementary file 1 [file JCMM-23-1257-s001.tif]

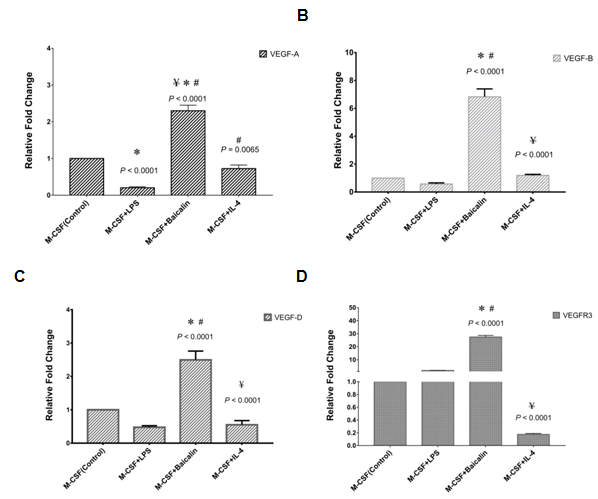

Supplement: Supplementary file 2 [file JCMM-23-1257-s002.tif]

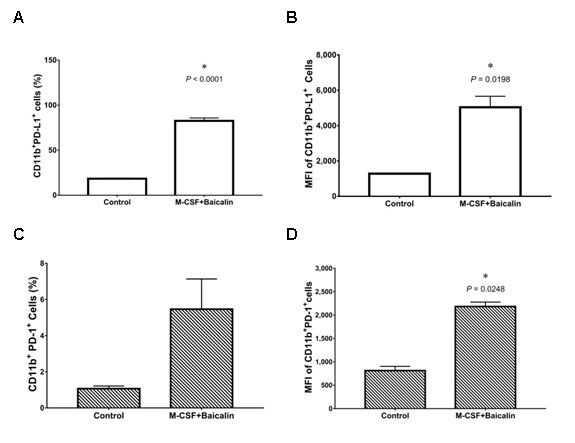

Supplement: Supplementary file 3 [file JCMM-23-1257-s003.tif]
